# Editorial: Discounting models in behavioral health economics and quantitative health psychology, volume II

**DOI:** 10.3389/fpubh.2023.1175519

**Published:** 2023-04-13

**Authors:** María José Muñoz Torrecillas, Salvador Cruz Rambaud, Taiki Takahashi

**Affiliations:** ^1^Department of Economics and Business, Mediterranean Research Center on Economics and Sustainable Development (CIMEDES), Universidad de Almería, Almería, Spain; ^2^Department of Behavioral Science, Center for Experimental Research in Social Sciences, Hokkaido University, Sapporo, Japan

**Keywords:** discounting models, behavioral economics, health economics, health psychology, public health

Behavioral economics can play a significant role in improving the healthcare systems by addressing some of the behavioral and psychological factors that affect patients' decision-making. We are interested on how time preferences and discounting affect individuals' health-related behaviors such as exercise, smoking, or medication adherence. For example, research has shown that individuals with a high discount rate are less likely to engage in preventive health behaviors such as following a healthy diet [i.e., (1–3)]. Research on time preferences and discounting related to health-related behaviors and policies to promote healthy behaviors is an active area of study.

This Research Topic, collects four articles of great interest in this area of study (two full length original research articles, one review article, and one brief research report article). Volume II completes the collection of articles published in “*Discounting models in behavioral health economics and quantitative health psychology*” [for an overview of the papers published in volume I, see (4)]. The papers included in volume II are summarized below.

Bibriescas et al. investigate the relationship between discount rates and health behaviors in a sample of ethnically diverse college students. Discount rates refer to the degree to which individuals prefer immediate rewards over delayed rewards. The authors hypothesized that individuals with higher discount rates would engage in riskier health behaviors, such as binge drinking, less testing for sexually transmitted infection (STI), and less exercise, compared to those with lower discount rates. The study was conducted using an online survey that measured discount rates and health behaviors in a sample of 395 college students from diverse ethnic backgrounds. Results show that higher social discount rates were associated with smaller likelihood to be tested for STIs, but not with smoking or poor exercising. Additionally, ethnicity was always a significant predictor for health behavior. These results suggest that discount rates may be a useful tool for identifying individuals who are at greater risk for engaging in risky health behaviors. The authors highlight the importance of considering ethnic differences in discount rates and health behaviors when designing interventions to promote healthy behaviors in an ethnically diverse population.

Brown and Stein explore the use of episodic future thinking (EFT) as a method to reduce delay discounting and maladaptive health behaviors. EFT is a technique that involves imagining and elaborating on positive future events to increase motivation and reduce the appeal of immediate rewards. The authors provide a comprehensive review of the literature on EFT and discuss the methodological considerations that researchers should take into account when using EFT in their studies. They also highlight the potential benefits of using EFT in public health interventions, particularly in the context of addressing maladaptive health behaviors. They conclude that EFT can be considered as a promising tool for promoting behavior change and reducing delay discounting, but further research is needed to determine the most effective ways to implement this technique in practice.

Krawiec et al. study the relationship between delay discounting of money and health outcomes and adherence to policy guidelines during the COVID-19 pandemic. This pandemic has led to many policy changes and recommendations aimed at controlling the spread of the virus. However, adherence to these guidelines has varied across individuals, and factors that influence adherence are not yet fully understood. A final sample of 338 individuals was used to complete a survey measuring their delay discounting tendencies for both money and health outcomes, as well as their adherence to COVID-19 policies such as wearing a mask and social distancing. They found no significant relationship between delay discounting for health outcomes and policy adherence. Nevertheless, they found strong evidence for the sign and the magnitude effects.

Yan et al. examine the relationship between trait impulsivity, choice impulsivity, and binge eating disorder (BED) in young adult students. Trait impulsivity refers to a general tendency to act impulsively across a range of situations, while choice impulsivity refers to the tendency to make impulsive decisions in specific contexts, such as food-related choices. The authors recruited 152 young adult students, and assessed their levels of trait impulsivity and choice impulsivity, as well as their likelihood of having probable BED. They found that participants with probable BED exhibited higher levels of trait impulsivity and choice impulsivity than those without probable BED. These findings suggest that both trait impulsivity and choice impulsivity may play a role in the development and maintenance of BED in young adults. Nevertheless, more research on this relationship is needed.

## Author contributions

All authors listed have made a substantial, direct, and intellectual contribution to the work and approved it for publication.
